# Motorcycle injury among secondary school students in the Tiko municipality, Cameroon

**DOI:** 10.11604/pamj.2016.24.116.5069

**Published:** 2016-06-02

**Authors:** Asonganyi Edwin Nyagwui, Namatovu Fredinah, Longho Bernard Che, Blomstedt Yulia

**Affiliations:** 1 Department of Epidemiology and Global Health, Umeå University, Sweden

**Keywords:** Awareness, practice, safety, accident, injury, students

## Abstract

**Introduction:**

Injury from motorcycle is a considerable cause of disability and death in the world and especially in low and middle-income countries; it is one of the most serious public health problems. In Cameroon, motorcycle is commonly used for transportation particularly among students. The aim of this paper is to study the risk-factors of the motorcycle-related accidents and injuries among secondary school students’ in the Tiko municipality, Cameroon.

**Methods:**

A cross sectional study was conducted in January 2012 on 391 students age 16-24 from public and private schools in the Tiko Municipality. Logistic regression was used to estimate the association between risk factors and injuries. A closed-ended and few open-ended questionnaire was used to collect data.

**Results:**

The study showed that over 70% of students used motorcycles always or often. Few had undergone any formal training for driving a motorcycle. The vast majority reported not wearing protective gear while driving or riding a motorcycle. Usage of protective gear was particularly low among girls. Over 16% reported using a motorbike always or occasionally under the influence of alcohol or drugs. Over 58% of respondents reported having an accident and over 35% were injured when driving or riding a motorcycle. Those who lived at the Tiko-Douala road have three times higher probability to sustain accidents and injuries than students residing elsewhere (OR 3.19 (1.20-8.46).

**Conclusion:**

It is deeply alarming that every second respondent in the study reported having been in an accident and every third motorcycle user was somehow injured. We therefore call for an immediate attention and a deeper investigation into the highlighted situation, particularly at Tiko-Douala road.

## Introduction

Injury from motorcycle is a considerable cause of death and disability in the world and is one of the most serious public health problems especially in low and middle-income countries (LMICs) where motorcycle is used purposely for transportation because of affordability and fast access to areas not pliable by motor vehicles [1]. It is estimated that 5.8 million people died world-wide from motorcycle injuries in 1998, corresponding to a rate of 97.9 per 100 000 population. Injury is the leading cause of death in all age groups, for both males and females and motorcyclists constitute a high proportion of fatalities in traffic crashes [1, 2]. Relative to four-wheeled cars, motorcycles are considered as greater risk of serious injury or death to operators and riders [3]. Motorcycling is risky and motorcyclists are 35 times more likely than passenger-car occupants to die in a motor car traffic crashes and 8 times more likely to be injured per vehicle-mile [4]. In Thailand, for example where the use of motorcycles are particular common and up to 80% of road traffic injuries patients resulted from motorcyclists [5].

A large number of motorcycle crashes and fatalities involve riders who lack a proper license or training, are speeding, and/or don't wear a safety helmet [6]. In LMICs, the failure to use motorcycle safety devices is often associated with inadequate education in safety issues and no law enforcement [7]. Thus effective interventions for prevention of motorcycle injuries are necessary and highly desirable particularly in these settings [8].

In Cameroon, the dwindling economy and the decay of infrastructure have led to the emergence of motorcycles for commercial transportation (popularly called “Okada”) over the past decade. In densely populated regions, where transportation poses a serious problem because of the limited number of public transportation and taxis, motorcycles have become a remedy, particularly for students. Many of school students of age 16-24 use motorcycles themselves or ride with commercial motorcycles to school daily. According to the laws of the Ministry of Transport in Cameroon, this age group is allowed to own and drive motorcycles, provided they have obtained appropriate education and use protective gears.

This study is the first one to explore the patterns of motorcycle usage among the secondary school students in Cameroon. The aim was to study the risk-factors of motorcycle-related accidents and injuries among this group in the Tiko municipality, Cameroon. The specific objectives were: to explore the practice of safety measures in motorcycle usage and to assess the risk factors and the prevalence of the motorcycle-related accidents and injuries among secondary school students in the Tiko municipality, Cameroon.

## Methods

### Study setting

This cross-sectional study was based in the Tiko municipality located in the Southwest Region of Cameroon with a total population of about 55914 [9]. It is coastal town with touristic sites and has industries, schools, business agencies. Its major towns are Mutengene, Likomba and Missellele [10]. English is a spoken language.

### Study population

The target populations for the study were high school students aged 16-24 years. The age group range was limited to those students that can use motorcycle themselves for transportation or those who use the commercial ones. Four high schools in this region were included in this study. Class registers were used to select students in each of the schools. Questionnaires were given to the students with odd number on the class register. In total 400 students were systematically recruited for the study.

### Data collection

Questionnaire consisting of close-ended and few open-ended questions English was used to collect data. The questionnaire was adapted from a questionnaire used in a similar study in London [11]. A pilot study was conducted to assess its validity. The updated questionnaire was used for data collection. Participants were given 15 minutes to respond to the questions, after which the questionnaires were collected back. Data were manually entered into Epidata-entry for analysis.

### Data analysis

The frequency and the prevalence of risk-factors for the motorcycle-related injuries were calculated as well as the prevalence of the motorcycle-related accidents and injuries in the studied population. Logistic regressions were used to calculate the association between the risk-factors and the motorcycle-related accidents and injuries in the sample. The results were presented as odds ratio with 95% CI. The software package in analyzing this data was STATA version 11.

#### Dependent variables

*Accident*(yes/no) derived from a question “Have you ever been in an accident when driving or riding a motorbike?”*Injury* (yes/no) derived from a question “Have you ever had received an injury as a result of a motorbike use (for example, when driving yourself or riding with someone)?”

#### Independent variables

Socio-demographic variables included *gender* (male/female), *age* (16-18; 19-21 and 22-24 years old), education (advanced; ordinary; first school leaving certificate), and *area of residence* which reflected different geographical regions within the Tiko municipality (Tiko, Likomba, Tiko-Douala road and Mutengene). Information on motorcycle usage was obtained through questions 5-9 and included *usage of motorcycle* (always; often; seldom/never), driving motorcycle (yes/no), ownership of a motorbike (yes/no). Knowledge of safety measures was studied by questions 9-16). *Motorbike training* was categorized as formal training, informal training, and no training at all. Answers to all questions on the usage of protective gear were grouped as always, occasionally and never. Information on accidents and injuries included *number of accidents* (1-2; 3-4; 5 or more times; never), *driving motorcycle* (yes/no) and an open-ended question on *wearing protective gear* when the accident occurred.

#### Ethics

The study was approved by the school authorities in all schools. All participants were informed about the study purpose and guaranteed anonymity and confidentiality. Participation was voluntary.

## Results

Response rate was 97.8%. There were slightly more boys than girls. The age group distributions were similar between boys and girls, with the majority of them being among the age group 19-21 years old (Table 1). Respondents who never used motorcycle were excluded from the further analysis (38 persons, 9.7%). Thus all further analyses were based on a sample of 353 students.

**Table 1:** Socio-demographic characteristics of students by sex (n=391)

Variables	Total (N=391)	Sex		P-Value
		Male (n=202)	Female (n=189)	
	N (%)	n (%)	n (%)	
***Age groups (Years)***				
16-18	134 (34.3)	69 (34.2)	65 (34.4)	0.259
19-21	189 (48.3)	92 (45.5)	97 (51.3)	
22-24	68 (17.4)	41 (20.3)	27 (14.3)	
***Education***				
Advanced Level	22 (5.6)	7 (3.5)	15 (8.0)	0.000
Ordinary Level	351 (89.8)	178 (88.1)	173 (91.5)	
First School Leaving Certificate	18 (4.6)	17 (8.4)	1 (0.5)	
***Residence***				
Tiko	227 (58.1)	107 (52.9)	120 (63.5)	0.161
Likomba	81 (20.7)	49 (24.3)	32 (16.9)	
Tiko-Douala Road	31 (7.9)	16 (7.9)	15 (8.0)	
Mutengene	52 (13.3)	30 (14.9)	22 (11.6)	
***How often do use motorcycle?***				
Always	121 (30.9)	60 (29.7)	61 (32.3)	0.153
Often	168 (43.0)	97 (48.0)	71 (37.6)	
Seldom	64 (16.4)	27 (13.4)	37 (19.6)	
Never	38(9.7)	18 (8.9)	20 (10.6)	

As shown in Table 1 73, 9% of respondents reported using motorcycle always or often (77.7% of boys and 69.9% of girls). Few reported owning a motorcycle (8.2%) and less than half of them reported receiving formal training, however 22.9% of respondents reported driving a motorcycle as shown in Table 2. The vast majority reported not wearing protective gear while driving or riding a motorcycle. Usage of protective gear was particularly low among girls. Over 16% reported using a motorbike always or occasionally under the influence of alcohol or drugs.

**Table 2:** Usage of motorcycle (n=353)

Variables	Total (N=353)	Sex		P-value
		Male (n=184)	Female (n=169)	
	N(%)	n(%)	n(%)	
***Motorcycle Ownership***				
No	324 (91.8)	159(86.4)	165(97.6)	0.000
Yes	29(8.2)	25(13.6)	4(2.4)	
***Motorcycle Training (among those who own motorcycle)***				
Formal training	13(44.8)	13(52.0)	0(0.0)	0.037
Informal training	12(41.4)	8(32.0)	4(100.0)	
No training at all	4(13.8)	4(16.0)	0(0.0)	
Missing values	324(-)	159(-)	165(-)	
***Do you drive motorcycle?***				
Yes	81(22.9)	72(39.1)	9(5.3)	0.000
No	272(77.1)	112(60.9)	160(94.7)	
***When on motorcycle how do you:*****Wear helmet?**				
Always	47(13.3)	31(16.9)	16(9.5)	0.001
Occasionally	56(15.9)	39(21.2)	17(10.1)	
Never	250(70.8)	114(61.9)	136(80.4)	
***Wear bright/reflective clothing?***				
Always	40(11.3)	27(14.7)	13(7.7)	0.054
Occasionally	113(32.0)	62(33.7)	51(30.2)	
Never	200(56.7)	95(51.6)	105(62.1)	
***Use daytime headlights?***				
Always	55(15.6)	35(19.0)	20(11.8)	0.052
Occasionally	80(22.6)	46(25.0)	34(20.1)	
Never	218(61.8)	103(56.0)	115(68.1)	
***Wear protective jacket?***				
Always	45(12.8)	31(16.9)	14(8.3)	0.001
Occasionally	77(21.8)	49(26.6)	28(16.6)	
Never	231(65.4)	104(56.5)	127(75.1)	
***Wear protective trousers?***				
Always	63(17.9)	41(22.3)	22(13.0)	0.050
Occasionally	100(28.3)	53(28.8)	47(27.8)	
Never	190(53.8)	90(48.9)	100(59.2)	
***Ride under the influence of alcohol/drugs?***				
Always	11(3.1)	8(4.3)	3(1.8)	0.369
Occasionally	47(13.3)	25(13.6)	22(13.0)	
Never	295(83.6)	151(82.1)	144(85.2)	

Over 58% of respondents reported having an accident when driving or riding a motorcycle at some point during the past years. The proportion of students who had injuries as result of accident was above 35%. The majority occurred when respondents were not driving a motorcycle, but using it as passengers (65.8% boys and 96.1% girls) and when none protective gear was worn (Table 3) Over 75.0% of the students said the cause of the accidents and injuries were due to human behaviors such as answering phone calls, high speed, overtaking, and inexperience while 25.0% of the students said the accidents and injuries were due to vehicles and environment e.g. bad road, bad tyres (not shown in a table). In the logistic regression males and females were analyzed together because of the small sample size. Nevertheless, few associations were significant, with the exception of residence areas. Those who lived in Tiko-Douala road were of higher likelihood in an accident as well as being injured from the accident, which remain significant even after adjustment for all explanatory variables. There was a higher likelihood of experiencing accidents when not wearing protective gear, driving when feeling tired or under the influence of drugs or alcohol, however, not significant (Table 4).

**Table 3:** The frequency of accidents and injuries (n=353)

Variables	Total (N=353)	Sex		P-value
		Male (n=184)	Female (n=169)	
	N(%)	n(%)	n(%)	
***Have an accident when driving or riding a motorcycle***				
No	147(41.6)	74(40.2)	73(43.2)	0.571
Yes	206(58.4)	110(59.8)	96(56.8)	
***How many times?***				
1-2 times	169(47.9)	86(46.7)	83(49.1)	0.358
3-4 time	32(9.1)	20(10.9)	12(7.1)	
> 5 times	5(1.4)	4(2.2)	1(0.6)	
Never	147(41.6)	74(40.2)	73(43.2)	
***Have you had an injury as a result of motorcycle***				
No	229(64.9)	111(60.3)	118(69.8)	0.062
Yes	124(35.1)	73(39.7)	51(30.2)	
	Total (N=124)	n(%)	n(%)	
***Were you driving?***				
*Yes*	27(21.8)	25(34.2)	2(3.9)	0.000
*No*	97(78.2)	48(65.8)	49(96.1)	
***What protection did you wear?***				
*Helmet/Jacket/trousers*	18(14.5)	16(21.9)	2(3.9)	0.005
*None of the above*	106(85.5)	57(78.1)	49(96.1)	

**Table 4:** Associationbetween accidents, injuries and motorcyclists’ background, and motorcycle usage among the students of Tiko Municipality (Odd Ratios (OR) with 95% CI)

	Accident		Injury	
	Uni-variate analysis	Multi-variate analysis+	Uni-variate analysis	Multi-variate analysis+
Variables	OR (95%CI)	OR (95%CI)	OR (95%CI)	OR (95%CI)
***Sex***				
Male	1 (Reference)	1 (Reference)	1 (Reference)	1 (Reference)
Female	0.88 (0.58-1.35)	1.20(0.72-2.01)	0.66 (0.42-1.02)	0.96 (0.56-1.65)
***Age***				
16-18	1 (Reference)	1 (Reference)	1 (Reference)	1 (Reference)
19-21	0.86 (0.54-1.38)	0.72 (0.43-1.20)	0.92 (0.56-1.49)	0.80 (0.47-1.36)
22-24	1.15 (0.61-2.17)	0.96 (0.48-1.90)	1.15 (0.61-2.16)	0.90 (0.45-1.81)
***Residence***				
Tiko	1 (Reference)	1 (Reference)	1 (Reference)	1 (Reference)
Likomba	0.94 (0.55-1.61)	0.80 (0.45-1.42)	0.96 (0.55-1.70)	0.79 (0.43-1.46)
Tiko-Douala Road	3.04(1.19-7.78)	3.19 (1.20-8.46)	2.73 (1.24-6.04)	2.52 (1.10-5.78)
Mutengene	1.39 (0.71-2.72)	1.30 (0.63-2.68)	0.72 (0.35-1.49)	0.62 (0.28-1.36)
***Do you drive?***				
Yes	1 (Reference)	1 (Reference)	1 (Reference)	1 (Reference)
No	0.37 (0.21-0.65)	0.31 (0.16-0.60)	0.33 (0.20-0.55)	0.33 (0.18-0.61)
***Wear protective clothing/helmet/boots***				
Yes	1 (Reference)	1 (Reference)	1 (Reference)	1 (Reference)
No	1.50 (0.98-2.30)	1.42 (0.90-2.23)	1.33 (0.85-2.06)	1.30 (0.81-2.08)
***Wear bright/reflective clothing?***				
Always	1 (Reference)	1 (Reference)	1 (Reference)	1 (Reference)
Occasionally	1.14 (0.55-2.35)	1.17 (0.54-2.53)	0.92 (0.44-1.93)	0.89 (0.40-1.96)
Never	1.42 (0.72-2.80)	1.65 (0.79-3.45)	0.72 (0.36-1.45)	0.79 (0.37-1.67)
***Drive while feeling tired***				
Never	1 (Reference)	1 (Reference)	1 (Reference)	1 (Reference)
Occasionally	1.44 (0.86-2.42)	1.69 (0.97-2.95)	1.19 (0.71-2.00)	1.34 (0.76-2.34)
Always	1.34 (0.67-2.67)	1.39 (0.65-2.99)	1.04 (0.52-2.11)	1.30 (0.59-2.86)
***Drive while under the influence of alcohol/drugs***				
Never	1 (Reference)	1 (Reference)	1 (Reference)	1 (Reference)
Occasionally	1.81 (0.93-3.52)	1.62 (0.79-3.34)	1.90 (1.02-3.53)	1.69 (0.86-3.35)
Always	0.92 (0.27-3.08)	1.26 (0.35-4.52)	0.44 (0.93-2.08)	0.60 (0.12-2.98)

+ This is adjusted for all other variables: sex, age, residence, do you drive, wear protective clothing/helmet/boots, wear reflective clothing, drive while feeling tired, drive while under the influence of alcohol/drugs.

## Discussion

This study revealed that the students’ practice of using motorcycle was a common phenomenon with 73.9% of respondents using motorcycle always or often. Nevertheless, the vast majority reported not wearing protective gear. In spite of existing laws and regulations, 70.8% of respondents reported never wearing helmet, 56.7% - never wearing reflective clothing. Moreover, over 16% reported using a motorbike always or occasionally under the influence of alcohol or drugs. Only half of those who owned a motorcycle had taken formal training in driving.

Not surprisingly, over 58% of respondents reported having been in an accident and 35% reported being injured as a result of an accident. The majority occurred when none protective gear was worn. While few associations were found significant in the logistic regression analysis, one stood out. Students who resided at Tiko-Douala Road had three times higher probability of being in an accident and 2.52 times higher probability of being injured in an accident than students residing elsewhere. This was significant even after association for all independent variables.

Tiko-Douala Road is a highway traffic area. It is possible that both speed and traffic intensity is higher there which makes it particularly dangerous for motorcyclists. Moreover, this highway traffic road has inadequate signs especially for crossing pedestrians. Busy highways, neglect of basic safety laws and regulations as well as lack of formal training in driving creates a dangerous combination for all traffic participants, particularly for those who are most exposed, i.e., motorcyclists. Young motorcycle users are at even higher risk. It has been repeatedly shown that the risks of motorcycle injury and death are highest among the young [12–15], even in the high-income countries as US [16]. This is often explained by their inexperience in operating a motorcycle [17]. It has also been shown that the risk of crash injuries is associated with experience, gender, distance ridden, and geographic region [18]. Due to a descriptive nature of this study and a small sample size, one cannot generalize the results for the whole Cameroon. Nevertheless, we argue that this study touches an important issue and revealed rather dramatic frequencies of accidents and injuries among students using motorcycles as a method of transportation to and from school. It is deeply alarming that every second respondent in the study reported having been in an accident and every third motorcycle user was somehow injured. We therefore call for an immediate attention and a deeper investigation into the highlighted situation, particularly at Tiko-Douala road.

The law and enforcement officials should implement the use of protective gears when using motorcycle. The motorcycle should be well controlled to ensure good state and maintenance. The Tiko-Douala road should be provided with a good pedestrian pavement and speed control signs for inhabitants’ resident in this area. Motorcycle riders should not use mobile phone while riding. Further studies are necessary to investigate other risk factors that may be associated to motorcycle accident and injuries. More important also is the exploration of the attitude of students regarding motorcycle usage.

## Conclusion

In conclusion, it is deeply alarming that every second respondent in the study reported having been in an accident and every third motorcycle user was somehow injured. We therefore call for an immediate attention and a deeper investigation into the highlighted situation, particularly at Tiko-Douala road.

### What is known about this topic

- Injuries are leading cause of death in developing countries and most specifically among young adults;
- Protective gears use has been shown to reduce death and disability among motorcyclists;
- Lack of training, speeding and inexperience of riding motorcycle may cause fatalities and crashes.

### What this study adds

- Most of the students don't wear helmet even if they are affordable and the study showed high risk of accidents and injuries at the Tiko-Douala road;
- Majority of the accidents and injuries which lead to death sometimes is due to human behaviors especially drunkenness, speeding, bad state of the motorcycle etc;
- Some of the students’ had recall bias especially the number of times and even the year they had motorcycle accidents and injuries.
